# Soluble Membrane Attack Complex: Biochemistry and Immunobiology

**DOI:** 10.3389/fimmu.2020.585108

**Published:** 2020-11-10

**Authors:** Scott R. Barnum, Doryen Bubeck, Theresa N. Schein

**Affiliations:** ^1^ CNine Biosolutions, LLC, Birmingham, AL, United States; ^2^ Department of Life Sciences, Imperial College London, London, United Kingdom

**Keywords:** complement, soluble membrane attack complex, sC5b-9, cholesterol-dependent cytolysins, MAC, diagnostics, sMAC

## Abstract

The soluble membrane attack complex (sMAC, a.k.a., sC5b-9 or TCC) is generated on activation of complement and contains the complement proteins C5b, C6, C7, C8, C9 together with the regulatory proteins clusterin and/or vitronectin. sMAC is a member of the MACPF/cholesterol-dependent-cytolysin superfamily of pore-forming molecules that insert into lipid bilayers and disrupt cellular integrity and function. sMAC is a unique complement activation macromolecule as it is comprised of several different subunits. To date no complement-mediated function has been identified for sMAC. sMAC is present in blood and other body fluids under homeostatic conditions and there is abundant evidence documenting changes in sMAC levels during infection, autoimmune disease and trauma. Despite decades of scientific interest in sMAC, the mechanisms regulating its formation in healthy individuals and its biological functions in both health and disease remain poorly understood. Here, we review the structural differences between sMAC and its membrane counterpart, MAC, and examine sMAC immunobiology with respect to its presence in body fluids in health and disease. Finally, we discuss the diagnostic potential of sMAC for diagnostic and prognostic applications and potential utility as a companion diagnostic.

## Introduction

The complement system is the most complex of the immunological and hematological pathways in human biology. Composed of ~50 proteins, four activation pathways (classical, lectin, alternative, and extrinsic) and a terminal lytic pathway, it is an important part of both innate and adaptive immune responses (1–3). Complement-mediated immune effector functions include chemoattraction of immune cells, activation of leukocytes, platelets and essentially all cell types proximal to complement activation, opsonization of invading pathogens, enhancement of the acute-phase response, lysis of susceptible pathogens and modulation of lymphocyte-mediated immune responses (1, 2, 4–6). Complement also serves to help in controlling T and B cell activation and function, stem cells and developmental processes, modulate basic cellular processes in intracellular sensing and cellular metabolism as it relates to immune responses (7–15), synaptic pruning (16, 17), modulation of the circadian clock (18), and possible contributions to schizophrenia (19, 20). Effector functions mediated by complement are driven by the proteolytic generation of activation fragments that either 1) bind to receptors expressed on both immune and non-immune cells, or 2) covalently attach to cell surfaces adjacent to sites of complement activation (1–3). These activities are tightly controlled by more than a dozen fluid-phase and membrane-bound regulatory molecules whose function is to keep complement activation in proportion to the amount of activator present and to limit damage to host tissues (1, 21, 22). Additional non-canonical roles of complement are discussed in a series published in Seminars in Immunology (23).

### sMAC: A Unique Activation Fragment

**Figure 1:**
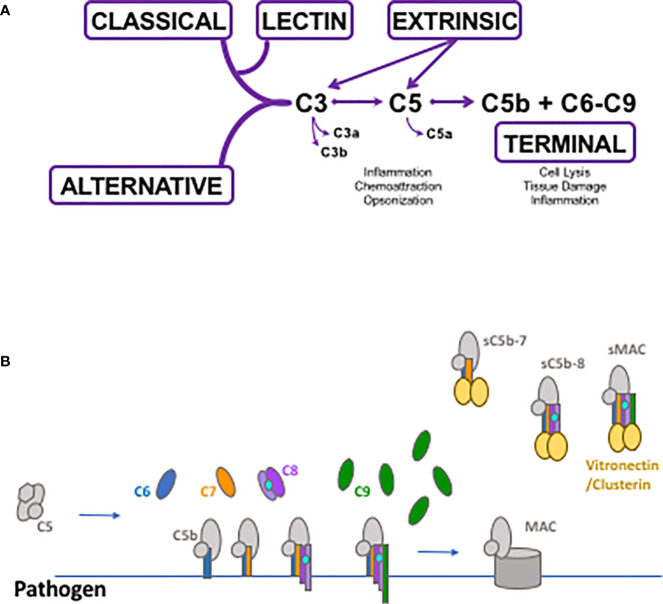
Schematic depicting complement activation and soluble membrane attack complex (sMAC) and MAC formation. **(A)** The classical, lectin and alternative pathways generate C3 and C5 convertases that cleave C3 and C5 into their biologically active fragments. Direct cleavage of C3 and C5 occurs through the extrinsic protease pathway which utilizes several enzymes of the coagulation system such as activated thrombin and plasmin and others. Activation through any of the pathways can generate C5b which initiates the formation of MAC and sMAC through the terminal pathway. **(B)** Schematic of MAC formation on a pathogen surface. Generation of C5b as a result of complement activation allows the non-covalent association of C6 through C9 and the production of the pore-forming membrane attack complex. Simultaneously with MAC formation, C5b in the fluid-phase can associate with C6 through C9 forming soluble intermediates leading to sMAC generation. All of the soluble intermediates and sMAC associate with vitronectin and/or clusterin preventing their insertion into pathogen or human cell membranes.

Activation of complement liberates more functional polypeptide fragments of various molecular species than any other immunological or hematological pathway. For example, activation of factor B releases Bb, a serine protease and key component of the alternative pathway C3 and C5 convertases, and Ba, a small polypeptide composed of three shushi domains with no known biological function. In contrast, cleavage of C3 and C4 generates C3a and C4a, respectively, which are small (~10 kDa) fragments that possess a wide range of functions including chemoattraction, antimicrobial activity, and modulation of T cell responses [reviewed in (6, 24)]. In addition, C3 and C4 cleavage produces multiple polypeptides from the larger ‘b’ fragments which are equally diverse in function (1, 2, 25). Enzymatic activity of complement serine proteases is responsible for production of at least a dozen activation fragments. The soluble membrane attack complex (sMAC) is an exception to the production of functionally-active polypeptide fragments. Generated by activation of the complement pathways, the formation of sMAC in the fluid-phase starts with the cleavage of C5 by C5 convertases, to C5a and C5b (**Figure 1A**). The addition of C6, C7, C8, C9 to C5b forms a basic MAC structure, which associates with the regulatory proteins clusterin and/or vitronectin, to form a soluble MAC complex inhibited from inserting into lipid bilayers (26–33). sMAC may have one to three C9 molecules and can bind one to two clusterin or vitronectin molecules, or a combination of clusterin and vitronectin molecules (**Figure 1B**). Thus, sMAC is not a single molecular species, but a family of closely related multi-molecular complexes. Based on this stoichiometry, at least fifteen different sMAC complexes are possible. Since each of the protein subunits in sMAC have polymorphic variants (34–43), there are many sMAC variants at the population level (similar to polymorphism at the population level for MHC molecules). The biological roles of these sMAC species in homeostatic conditions and disease pathophysiology are undefined. In contrast, studies in recent years have demonstrated that the MAC contributes to intracellular signaling, inflammation, and other functions (44–47).

**Figure 2:**
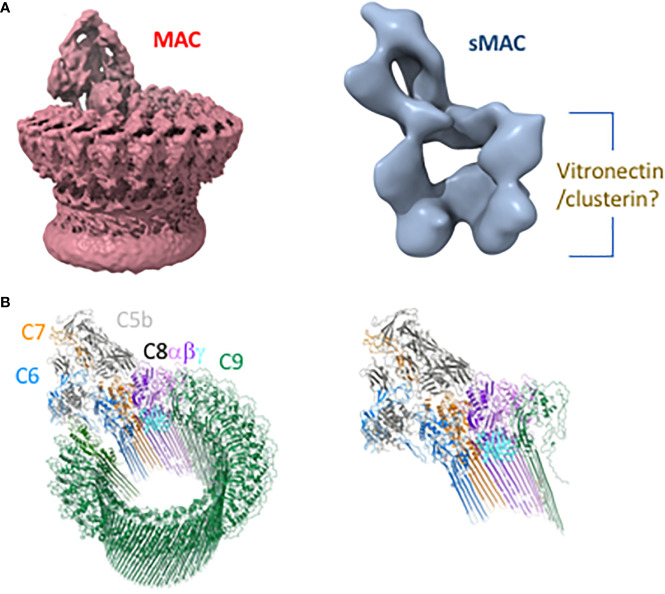
Structures of membrane attack complex (MAC) and soluble MAC (sMAC). **(A)** CryoEM reconstruction of MAC at 4.9 Å resolution (EMD-0110) (53) shows a ring-like arrangement of complement proteins that comprise MAC (left). By contrast, the cryoEM reconstruction of sMAC at 24 Å resolution (EMD-1991) (33) reveals that the ring is stopped short by chaperones vitronectin and clusterin to form an arc (right). Vitronectin and clusterin may act to cap the arc and/or bind exposed hydrophobic residues of unfurled beta-hairpins. **(B)** The left depicts the MAC (PDB-6H04) as a ribbon diagram in which complement proteins are colored: C5b (gray), C6 (blue), C7 (orange), C8β (dark purple), C8α (light purple), C8γ (cyan), and C9 oligomer (green). On the right is a single copy of each protein that may fill the arc of density depicted in the low-resolution sMAC structure.

Several intermediates leading to the formation of sMAC and MAC have been well characterized biochemically (**Table 1**). Cryo electron microscopy structures of sMAC (33) and MAC (33, 52, 53) suggests a similar overall arrangement of complement proteins within the complex (**Figure 2A**). In both complexes, C5b serves as a structural scaffold that organizes C6, C7, C8 and C9 into an arc through their pore-forming membrane attack complex perforin (MACPF) domains. During MAC formation, the core MACPF domains of C6, C7 C8 and C9 undergo a dramatic structural rearrangement in which two helical bundles unfurl to form a pair of β-hairpins that insert into the lipid bilayer. While it remains unclear from the low-resolution sMAC structure if these transmembrane-hairpins domains have unfurled, both complexes are of a similar length suggesting that at least some of sMAC β-hairpins maybe extended (**Figure 2B**). Negative stain electron microscopy images of vitronectin-labeled sMAC suggest that chaperones may bind to exposed hydrophobic hairpins (29), however, the molecular details of how clusterin and vitronectin prevent membrane insertion of sMAC are still unresolved. In MAC, the helix-to-hairpin transition of membrane-interacting residues exposes a charged surface of the MACPF. Charge complementarity between MACPF-MACPF interfaces is one of main factors that determines the direction of MAC assembly, and likely plays a similar role in formation of sMAC (53). The non-pore forming domains of complement proteins act as regulatory auxiliary modules, preventing the premature release of transmembrane β-hairpins during MAC assembly. How these regulatory domains are oriented in sMAC remains to be seen. High resolution structures of inhibited MAC complexes will be necessary to understand how regulators, such as vitronectin and clusterin, block membrane association of MAC.

**Table 1:** Physicochemical parameters of soluble membrane attack complex (sMAC) and related complexes.

sMAC complex	Subunit composition	Mol. Wt.	Sedimentation coefficient (S)	Reference
sC5b-9	C5b C6, C7, C8, C9 (1 each), clusterin and/or vitronectrin	~1 MDa	23	(29)
C5b-6	C5b, C6*	328 kDa	11.5	(48)
sC5b-7	C5b, C6, C7, vitronectin or clusterin**	668 kDa	18.5–20	(49)
sC5b-8	C5b, C6, C7, C8, vitronectin, and/or clusterin**	800–850 kDa	19 – 21	(50)
MAC	C5b C6 C7 C8 (1 each), C9 (up to 18), vitronectin and/or clusterin	1.6 MDa	33	(26, 51)

*Studies have shown that vitronectin inhibits lytic activity of C5b,6, but no tri-molecular complex containing vitronectin has been characterized.

**The precise number of clusterin or vitronectin subunits binding to sC5b-6, sC5b-7, and sC5b-8 is currently unknown.

### sMAC in Biological Fluids in Normal and Disease States

sMAC was first quantitated in plasma in healthy individuals almost 50 years ago (54). Numerous studies since that time have documented the presence of sMAC in most body fluids as discussed below. Although sMAC is present in many of these fluids, the mechanism(s) that generate tissue-specific, basal sMAC levels have received little attention. The continuous activation of complement at low levels through C3 tickover, first described in the early 1970s (55, 56), likely contributes to sMAC generation. In addition to tickover, it is well established that the coagulation system, like complement, is continuously active at a basal level, and thus basal activation of the coagulation and fibrinolytic systems may also contribute to sMAC generation (57–61). This mechanism of complement activation is known as the extrinsic pathway, and it bypasses convertase formation and directly activates C3 and C5 [reviewed in (62, 63)]. The relative contribution of each of these mechanisms and any others that may be involved in basal sMAC generation remains to be established. Once complement is activated however, the high plasma concentration of vitronectin (200–400 μg/ml, (64) and clusterin [150–540 μg/ml, (65)] relative to C9 [~50–60 μg/ml, (66)] suggests formation of sMAC is favored, particularly since both regulatory proteins are elevated in concentration in the acute phase response (67, 68). Mathematical modeling supports this possibility revealing that sMAC is generated rapidly on activation of the classical and alternative pathways reaching peak concentration within 15 min (69). In contrast, MAC production and deposition on pathogens surfaces is characterized by a lag phase of ~20 min, followed by rapid production and deposition that peaks after 50 min (70).

Timing is important for the formation of functional MAC pores and could tip the balance to sMAC production. Rapid over-activation of the complement terminal pathway may overwhelm the C5 convertase. In addition to the proteolytic cleavage of C5, the C5 convertase also plays an important role in orienting MAC assembly precursors at the membrane (71). Improperly inserted precursors could then be scavenged by fluid-phase vitronectin and clusterin, to produce sMAC. On a bacterial surface, convertase generated C5b6 must rapidly recruit C7 to form functional MAC (72). If there is a delay in availability of C7, the inert C5b7 complex could be scavenged by clusterin and vitronectin. Indeed, C5b7 is the first MAC intermediate to bind these two chaperones.

#### Plasma and Serum

Although sMAC was detected in plasma several decades ago (54), the first quantitative assays based on sMAC neo-epitopes were not developed until the mid-1980’s (73, 74). sMAC levels of plasma or serum were frequently measured with in-house assays using serum activated zymosan or inulin as a standard control. These standards and assays were not well characterized and interpretation of the results were complicated by variable assay sensitivity, sample handling, and sample storage (73–77) as highlighted in recent study by Yang and colleagues (78). The International Complement Standardization Committee has since defined an activation standard for quantitation of complement activation products, including sMAC, termed International complement standard #2 (79). International complement standard #2 was derived from healthy donor-derived pooled serum activated with heat-aggregated IgG and zymosan. The utility of this standard (defined as complement activation units, CAU) is limited by variability in pooled serum between different donor cohorts and the reagents used to activate complement. The relationship of CAU to standard measures of protein concentration remains poorly defined. However, immunoassays using purified sMAC as a reagent for generating quantitative standard curves overcomes these limitations.

A recent study using a sMAC ELISA (Quidel, Corp., San Diego, CA) reported mean plasma and serum levels of sMAC of 121 ± 3.7 ng/ml (n=199) and 175 ± 8.1 ng/ml (n=49) respectively, in healthy adult donors (80). Plasma sMAC levels were similar between African-Americans and Caucasians and between males and females. Interestingly, plasma sMAC levels in individuals above 50 years of age were significantly higher than those in their 40’s and younger. The levels of sMAC in neonates have been measured in cord blood plasma samples obtained immediately after birth and were markedly lower than adult levels (81). This study, and a number of others, have shown that terminal pathway proteins comprising the MAC (C6-C9) are significantly lower in pre-term and full term infants, as are proteins of the classical and alterative pathways [reviewed in (82)]. In addition, a recent study determined that the blood levels of C9 in children less than one year of age were significantly lower compared to adults, and adult levels were reached between two and eighteen years of age (83). These studies indicate that sMAC levels in are lower in children, in part, because the concentration of proteins that compose the MAC are lower. Nonetheless, sMAC levels in children increase during infection, and activation of complement in neonatal serum by cobra venom factor also increased sMAC levels (81). Additional studies to determine sMAC blood levels in healthy adults and children are warranted to determine the value of sMAC as a diagnostic and prognostic tool in disease settings.

The sMAC in plasma and serum has been measured in many clinical settings including infectious and autoimmune disease, transplantation, trauma, and complement deficiencies (**Table 2**). The level of sMAC increases in these conditions in a disease-dependent fashion. However, an encompassing generalization regarding the magnitude and kinetics of the responses is not possible due to the variability in assay types used to quantify sMAC, and the baseline differences of sMAC concentration between serum and plasma. For this reason, we have not included the level of sMAC for the diseases and conditions listed in **Tables 2**–**4**. *In vivo* studies in rabbits have demonstrated that sMAC is eliminated with a half-life of 30–50 min (170), but no half-life studies have been reported for human sMAC. It is clear the diagnostic and prognostic value of sMAC in blood requires assay and sample handling standardization, particularly as complement therapeutics move into the clinical treatment repertoire (171).

**Table 2:** Soluble membrane attack complex (sMAC) changes in blood, plasma, and serum in various clinical conditions.

Body fluid	Clinical setting	Reference
Blood	Infectious disease: Pneumoniae HIV Systemic meningococcal infection Sepsis Dengue shock syndrome Malaria	(80, 84–90)
	Autoimmune disease: Arthritis Lupus ANCA-associated vasculitis Anti-phospholipid syndrome Multiple sclerosis Neuromyelitis optica Myasthenia gravis C3 nephritic factors (immune complex-membranoproliferative glomerulonephritis)	(91–105)
	Complement deficiency/mutations: PNH aHUS CFHR3/1 AMD TTP	(106–112)
	Transplantation/ECMO: Heart Lung Kidney/dialysis Autologous stem cells Red blood cells Transplant-associated thrombotic Microangiopathy	(113–118)
	Trauma	(119, 120)
	Dialysis & related treatments: Hemodialysis Peritoneal dialysis Intravenous iron treatment	(121–125)
	Cardiac failure/disease	(126–128)
	Psychiatric disorders: Bipolar disorder	(129)

**Table 3:** Soluble membrane attack complex (sMAC) changes in cerebrospinal fluid (CSF) in various clinical conditions.

Body fluid	Clinical setting	Reference
CSF	Infectious disease: Bacterial/cryptococcal meningitis Intraventricular shunt infections	(80, 130–134)
	Autoimmune disease: Multiple sclerosis Neuromyelitis optica Clinically Isolated syndrome Guillain–Barré syndrome Sjogren’s syndrome Systemic lupus erythematosus	(91, 135–141)
	Traumatic brain injury	(142, 143)
	Subarachnoid hemorrhage	(144)
	Alzheimer’s disease	(145)

**Table 4:** Mucosal and synovial soluble membrane attack complex (sMAC) changes in various clinical conditions.

Body fluid	Clinical setting	Reference
Urine	Autoimmune disease: Diabetic nephropathy ANCA-associated glomerulonephritis	(146)
	Kidney disease: Membranous nephropathy Acute tubulointerstitial nephritis Diabetic nephropathy Focal segmental Glomerulosclerosis Acute post-streptococcal Glomerulonephritis	(147–152)
	Transplantation	(153–155)
	Preeclampsia	(156)

Synovial Fluid	Arthritis	(92, 94, 157)
Pleural Fluid	Tuberculosis Rheumatic disease Malignancy Dengue shock syndrome	(88, 158–163)
Peritoneal Fluid/Ascites	Endometriosis acute pancreatitis	(158, 164–166)
Pericardial Fluid	Pericarditis	(158)
Burn Bullae (Blister) Fluid	Burn injury	(158)
Ovarian Follicular Fluid	Infertility	(167)
Seminal Plasma	Infertility	(168)
Aqueous Humor	Glaucoma	(169)

#### Cerebrospinal Fluid

The normal range of sMAC concentration in cerebrospinal fluid (CSF) of healthy individuals has not been established, in part, because of the clinical risk and discomfort surrounding procuring CSF *via* lumbar puncture. As a result, normal levels of sMAC in CSF have frequently been derived from cohorts with “other neurological diseases” or from patients who underwent lumbar puncture as a part of standard clinical care and had negative bacterial cultures. In most studies using the Quidel sC5b-9 ELISA, CSF sMAC levels range from undetectable to the low nanogram/ml range (10–20 ng/ml) (130, 142, 144, 172, 173). Studies analyzing the sMAC CSF/serum quotient using Reiber–Felgenhauer nomograms of IgM suggest that sMAC is intrathecally produced rather than diffusing across the blood brain barrier (BBB) as has been shown for C9 (173, 174). There are exceptions to this low normal range. For example, Aly and colleagues reported mean levels of sMAC CSF to be ~50 ng/ml in neonates with hypoxic-ischemic encephalopathy (175). The reasons for this higher level are unclear, but may be due to developmentally-reduced integrity of the BBB shortly after birth, to the elevated level of plasma proteins found in neonatal CSF compared to adults, or to the transport of plasma proteins across choroid plexus epithelial cells in fetal and neonatal brain [reviewed in (176)]. It is also possible that CSF sMAC in neonates is generated as a result of complement-mediated synaptic pruning (16) during neurodevelopment, which is subsequently cleared postnatally. Other studies have reported sMAC concentrations in control groups range from high ng/ml to low μg/ml levels (131, 135, 136). Although non-standardized, in-house sMAC assays were used many of these studies, one likely reason for the high sMAC levels in the control groups was the inclusion of patients with tumors, Huntington’s disease, stroke, seizure disorder, cerebellar degeneration, progressive supranuclear palsy, or undetermined infections, which are, at least in part, inflammatory in nature.

Despite the contrasting reports on the levels of CSF sMAC in healthy individuals, it is clear the levels increase in a number of pathological conditions. **Table 3** lists a number of neurological diseases in which sMAC increases relative to levels in other neurological diseases. In bacterial meningitis and shunt infections, sMAC levels have been reported to increase compared to uninfected controls (130, 132–134). In shunt infections, the increase in sMAC was remarkably high (over 100-fold) compared to control CSF (130). Similar dramatic changes in sMAC levels have been reported for traumatic brain injury (as high as 1,800-fold) and subarachnoid hemorrhage (~200-fold) compared to control CSF (142–144). sMAC concentration of this magnitude in CSF suggests its production is derived through multiple mechanisms and sources including: 1) increased intrathecal complement production and activation, 2) blood-derived sMAC leaking across a compromised BBB, and 3) *in situ* generation of sMAC at injury site(s). Interestingly, admixture experiments using human CSF and serum demonstrated that sMAC could be generated in a dose-dependent fashion (up to 5-fold over CSF alone) (144). However, there are cases where sMAC levels are not elevated in infectious or other pathological conditions. For example, in viral and fungal infections, CSF sMAC levels do not increase or increase minimally (131). In idiopathic normal pressure hydrocephalus, a disorder characterized by faulty CSF mechanical dynamics and associated neurodegeneration and inflammation (177), median sMAC levels were a low ~13 ng/ml (173). The reason for the differences in sMAC levels in these latter pathological conditions is unclear, but if verified by additional studies, they could provide differential diagnostic opportunities.

In central nervous system, autoimmune diseases such as multiple sclerosis (MS), Guillain-Barre syndrome, Sjogren’s syndrome and systemic lupus erythematosus, there have been reports of increased levels of sMAC (91, 135–138). However, other MS studies have reported no increases in sMAC (139, 178). Studies also present conflicting findings for sMAC levels in neuromyelitis optica (91, 139, 178). Clinically isolated syndrome, a neurodegenerative disease reminiscent of MS (179), has also been examined for changes in CSF sMAC levels. Although sMAC has been detected, the levels do not appear to increase in a clinically meaningful way, but the number of studies is limited (140, 141). There are several other central nervous system diseases where the MAC contributes to disease pathogenesis and, by extension, sMAC levels may change during the course of disease progression. These include epilepsy (180), Parkinson’s disease (181) amyotrophic lateral sclerosis (182), Alzheimer’s disease (183), various psychiatric conditions (20, 184) and possibly autoimmune encephalitis (185, 186). The inconsistencies noted in some of the above-mentioned studies most likely stem from the use of different sMAC assays, differential sample handling and storage, and the rarity of healthy patient CSF as a negative control. Going forward it would be important to agree on a standard assay for quantitating sMAC and to adopt standardized protocols for handling CSF samples such as that employed by the BioMS-eu network (187, 188).

#### Urine

Most complement proteins are too large to be excreted in urine. Even factor D, the smallest of the complement proteins (~ 24 Kd), does not pass through the tubular epithelium unless there is a kidney defect (189, 190). With a molecular weight approaching 1 MDa (29), studies have suggested that urinary sMAC is most likely locally generated rather than transported from blood into urine (147, 148, 153, 191). A number of factors may contribute to local sMAC generation in the kidney including high levels of proteinuria, cellular debris, urinary ammonia, and low urinary pH (153). In healthy individuals, sMAC is generally undetectable in urine regardless of the type of assay employed. The kidney appears particularly susceptible to complement-mediated damage for a variety of reasons [reviewed in (192)]. Not surprisingly then, nearly all studies examining for sMAC in urine are derived from patients with a variety of acute and chronic kidney diseases or post-kidney transplantation (147–155) (**Table 4**). A number of urinary biomarkers have been identified for acute kidney injury including kidney injury molecule-1 (KIM-1), IL-18, and others (193). Recent studies suggest that sMAC urinary levels are diagnostic for interstitial inflammation in acute kidney injury associated with nephritis (150) and severe preeclampsia (156) particularly in combination with KIM-1.

Surprisingly there is little known regarding sMAC and urinary tract infections (UTI). It has been shown that C3 promotes colonization of the upper urinary tract by *E. coli* and that C3- and C4-deficent mice develop fewer renal infections (194). Furthermore, in animal studies, C5a appears to exacerbate UTI through enhancing inflammation and recruitment of leukocytes as C5aR-deficient mice had less renal injury and reduced bacterial load compared to wild type mice (195). These studies indicate that complement contributes to UTI, at least for some pathogens, and that the terminal pathway could be involved since C5a is generated. It would be worth determining baseline levels of sMAC (once assays with higher sensitivity have been developed) in the urine of healthy individuals and comparing it to the levels in UTI patients. sMAC might be an easy biomarker to monitor in UTI, especially in chronic pyelonephritis.

#### Synovial and Mucosal Fluids

In addition to blood, CSF, and urine, sMAC is found in synovial, pleural, pericardial and peritoneal fluid under conditions of infection, malignancy, or autoimmune disease (listed in **Table 4**). These fluids are routinely collected for diagnostic purposes, primarily to identify bacterial or viral infections, as well as other medical conditions (196–201). Less commonly analyzed is blister fluid from burn patients. Blister fluid is receiving more attention as a possible diagnostic tool based on recent biochemical and proteomic studies [reviewed in (202, 203)]. sMAC and other complement activation proteins have been detected in blister fluid, however their diagnostic utility remains to be determined (158). Complement components are present in male and female reproductive systems and play a role in both fertility and infertility (204, 205). The presence of sMAC in ovarian follicular fluid and seminal plasma not only indicates complement activation, but suggests possible complement-mediated contributions to infertility (204). This remains an understudied topic and is worth pursuing given the general worldwide decline in fertility (206). sMAC has also been detected in aqueous humor of patients with exfoliating glaucoma (169), but not in patients with neovascular age-related macular degeneration (207). sMAC may be present in other body fluids such as tears, nasopharynx secretions, intestinal secretions, and gingival crevicular fluid, but these have not yet been reported. Support for this possibility comes from studies demonstrating the presence of C5a in normal tears and aqueous humor from patients with cataracts, glaucoma, anterior uveitis, or gingival crevicular fluid (208–210).

### sMAC in Complement Diagnostics

Changes in the blood levels of either complement proteins or complement functional activity have served as a valuable diagnostic tool for autoimmune diseases, syndromes, and complement deficiencies for over 60 years [initially reviewed in (211)]. Since then our understanding of the complement system and its relationship to the pathophysiology of infectious and autoimmune disease has increased significantly, and most clinical laboratories routinely run at least some complement-related diagnostic assays (212–214). In addition, commercial diagnostic laboratories offer an extensive array of assays to quantitate blood levels of many complement proteins, measure overall complement function, assess pathway- and protein-specific function and identify auto-antibodies to complement proteins. Identifying complement genetic mutations that contribute to syndromes such as hereditary angioedema, hemolytic uremic syndrome, and rare variants that contribute to deficiency or dysfunction [reviewed in (215)], is now offered by some diagnostic laboratories. The value of complement diagnostics will continue to grow as understanding of the role of complement in autoimmune, infectious, psychiatric diseases, and malignancies expands in the coming years (17, 216–218).

Although sMAC levels in blood, CSF, and other body fluids have been studied as a possible biomarker for diseases and inflammatory conditions (**Tables 2** **–** **4**), those studies have not translated into common use of sMAC as a clinical diagnostic tool. The literature provides numerous examples of the utility of sMAC as a diagnostic biomarker, but the lack of comprehensive reviews on this topic may be one contributing factor to the under-appreciation of its potential. There is no evidence to suggest that intermediates on the way to sMAC formation (sC5b-7 and sC5b-8) have any diagnostic value and there are currently no assays to specifically measure these MAC-related complexes. The advent of complement therapeutics may, however, be a game-changer for sMAC as a diagnostic tool. The anti-C5 antibody eculizumab prevents MAC and sMAC formation by blocking the cleavage of C5 into C5a and C5b, thereby inhibiting the terminal pathway (219). Initially used for treatment of patients with paroxysmal nocturnal hemoglobinuria and atypical hemolytic uremic syndrome, eculizumab has more recently been used in the management of myasthenia gravis, antibody-mediated graft rejection, neuromyelitis optica, and other conditions (220). Several studies have demonstrated that sMAC levels correlate well with eculizumab dosing further indicating that sMAC may be a useful biomarker for monitoring dosing and also aid in developing personalized patient treatment plans. This would usher in a new era in complement diagnostics particularly if patients could measure sMAC (and/or other complement fragments) at home and relay the information directly to their physician or clinic. This could include patients being treated for paroxysmal nocturnal hemoglobinuria and atypical hemolytic uremic syndrome (106, 221, 222), age-related macular degeneration (107), glomerulonephritis (223), hematopoietic stem cell transplantation (transplant associated thrombotic microangiopathy) (224), thrombotic thrombocytopenia purpura (108, 225), and acute post-infectious glomerulonephritis (226). sMAC monitoring may also have diagnostic value in anti-TNF-α treatment of spondylarthropathies (227), indicating the diagnostic value of sMAC exists beyond complement-specific therapeutics. By extension, sMAC may have diagnostic value in monitoring treatment in rheumatoid, psoriatic arthritis, and other autoimmune diseases given the findings in spondylarthropathies. Complement therapeutic drugs that target the terminal pathway directly or that inhibit the alternative pathway [through which most MAC/sMAC is generated (228)] are currently in development, and it may be beneficial to use sMAC as a biomarker in companion diagnostics to monitor drug efficacy and help manage patient dosing.

## Conclusion

The terminal complement pathway gives rise to the MAC and multiple sMAC isoforms. Although multiple immunological roles have been identified for the MAC, little is known regarding the immunobiology of sMAC and intermediates generated during the formation of sMAC. There is, however, a large body of preclinical and clinical studies suggesting that sMAC may be a valuable diagnostic tool in multiple disease settings. In order to fully appreciate the diagnostic potential of sMAC, a number of points should be addressed going forward. These include:

- Assay standardization for quantitating sMAC to allow comparison between datasets and disease settings
- Sample handling and storage standardization to maximize sample stability
- Increased reliance on true healthy controls instead of “non-inflammatory” or “other disease” control sample sets
- Studies to determine basal sMAC fluid levels across multiple demographics

In addition to formalized standardization, there is still much we do not know regarding sMAC with respect to basic physiology and biology. For instance, does sMAC containing vitronectin mediate unknown complement functions or contribute to hematological or cancer-related functions? The multi-functional roles of clusterin and vitronectin may provide insight into sMAC immunobiology, including identification of receptors used in the course of sMAC turnover. These would further aid in the use of sMAC as a biomarker for disease.

## Author Contributions

Conception and design was by TS and SB. Writing, review, and revision of the manuscript was performed by TS, DB, and SB. All authors contributed to the article and approved the submitted version.

## Funding

This work was supported in part by a grant from the National Institutes of Health (R43-AI132038) to TS and SB. This project received funding from the European Research Council (ERC) under the European Union’s Horizon 2020 research and innovation programme (grant agreement No. 864751 to DB).

## Conflict of Interest

SB and TS are co-founders of CNine Biosolutions, LLC, a biotech company developing complement diagnostics and co-inventors on United States patents # 10,535,004 and 10,630,774 and European Union patent # 3137908, both entitled, *Methods and Compositions for Diagnosis and Treatment of Meningitis.*

The remaining author declares that the research was conducted in the absence of any commercial or financial relationships that could be construed as a potential conflict of interest.
